# Transcriptional responses in developing lesions of European common ash (*Fraxinus excelsior*) reveal genes responding to infection by *Hymenoscyphus fraxineus*

**DOI:** 10.1186/s12870-020-02656-1

**Published:** 2020-10-06

**Authors:** Shadi Eshghi Sahraei, Michelle Cleary, Jan Stenlid, Mikael Brandström Durling, Malin Elfstrand

**Affiliations:** 1 Department of Forest Mycology and Plant Pathology, Swedish University of Agricultural Sciences, Uppsala, Sweden; 2 Department of Ecology and Genetics, Uppsala University, Uppsala, Sweden; 3 Southern Swedish Forest Research Center, Swedish University of Agricultural Sciences, Alnarp, Sweden

**Keywords:** Ash dieback, *Fraxinus excelsior*, HMGR1 (3-hydroxy-3-methylglutaryl-coenzyme a reductase 1), ATAF1, Resistance, Necrotic lesion

## Abstract

**Background:**

With the expanding ash dieback epidemic that has spread across the European continent, an improved functional understanding of the disease development in afflicted hosts is needed. The study investigated whether differences in necrosis extension between common ash (*Fraxinus excelsior*) trees with different levels of susceptibility to the fungus *Hymenoscyphus fraxineus* are associated with, and can be explained by, the differences in gene expression patterns. We inoculated seemingly healthy branches of each of two resistant and susceptible ash genotypes with *H. fraxineus* grown in a common garden.

**Results:**

Ten months after the inoculation, the length of necrosis on the resistant genotypes were shorter than on the susceptible genotypes. RNA sequencing of bark samples collected at the border of necrotic lesions and from healthy tissues distal to the lesion revealed relatively limited differences in gene expression patterns between susceptible and resistant genotypes. At the necrosis front, only 138 transcripts were differentially expressed between the genotype categories while 1082 were differentially expressed in distal, non-symptomatic tissues. Among these differentially expressed genes, several genes in the mevalonate (MVA) and iridoid pathways were found to be co-regulated, possibly indicating increased fluxes through these pathways in response to *H. fraxineus*.

Comparison of transcriptional responses of symptomatic and non-symptomatic ash in a controlled greenhouse experiment revealed a relatively small set of genes that were differentially and concordantly expressed in both studies. This gene-set included the rate-limiting enzyme in the MVA pathway and a number of transcription factors. Furthermore, several of the concordantly expressed candidate genes show significant similarity to genes encoding players in the abscisic acid- or Jasmonate-signalling pathways.

**Conclusions:**

A set of candidate genes, concordantly expressed between field and greenhouse experiments, was identified. The candidates are associated with hormone signalling and specialized metabolite biosynthesis pathways indicating the involvement of these pathways in the response of the host to infection by *H. fraxineus*.

## Background

Since the 1990’s a new alien invasive disease affecting common ash (*Fraxinus excelsior* L.) trees has caused severe dieback and mortality across Europe [1]. The causal agent, *Hymenoscyphus fraxineus* Baral, Queloz, Hosoya has spread across almost all the natural range of common ash in Europe representing a major threat to this important tree species and associated biodiversity [1, 2]. Over the last decade there has been great leaps in the understanding of the ongoing ash dieback epidemic. The use of DNA-based markers and whole genome sequencing have given insights into the origin of *H*. *fraxineus* and its evolutionary potential [3–5]. Similarly molecular genomics, metabolite profiling, genome and transcriptome sequencing has contributed understanding of the genetic diversity and level of resistance to *H. fraxineus* in the European ash population in the wake of the devastating epidemic [6–10]. However, the understanding of the interaction between common ash and *H*. *fraxineus* is still quite limited and this may hamper attempts to control the ash dieback epidemic using genetic selection. To gain functional understanding of an interaction between a tree species and its pests and pathogens the molecular responses of the interaction can be analysed. Molecular responses can be defined as production of distinct specialized metabolites [8, 9, 11] or transcripts from expressed genes [6, 12, 13]. Specialized metabolites have been relatively well studied in the interaction between *F. excelsior* and *H. fraxineus* showing for instance the importance of various phenolic, terpenoid derivatives and plant hormones in control of infection and symptom development [7–9, 14]. The potential to select superior genotypes based on metabolite profiles has been explored [11].

The functional understanding of the mechanisms underlying variation in eg. metabolite profiles and metabolite accumulation patterns between trees with varying susceptibility is still limited in forest trees and common ash is no exception. Plant biotic stress interactions have been dissected in herbaceous model plants which has permitted opportunities to describe and exploit the plant defence systems, involving both pattern triggered immunity (PTI) and effector triggered immunity (ETI), as well as multiple plant hormone signalling pathways [15, 16]. A challenge in forest trees is that the underlying resistance mechanisms are not always the same as described or predicted in model systems, necessitating direct study of defence pathways and strategies utilized by tree species [17]. Analyses of the transcriptional regulation in for instance comparisons of inoculations and mock treatments, or of resistant and susceptible accessions of plants, have allowed for the identification of genes conferring resistance to pests and pathogens, including genes involved in pathogen perception, signaling and specialized metabolite biosynthesis pathways [13, 18–21].

In this study we performed controlled inoculations of trees with well-known levels of susceptibility with a single isolate of *H. fraxineus* and RNA sequenced tissue samples to examine the transcriptional responses in *F. excelsior* to infection by *H. fraxineus*. The study addressed the following hypotheses: (i) differences in necrosis extension between trees with different levels of susceptibility have different gene expression patterns both in non-symptomatic and symptomatic tissues; (ii) the transcriptional responses explain the phenotypic response (length of necrosis); (iii) the transcriptional responses provide an insight into the mechanisms leading up to necrosis formation. In association with the third hypothesis, we performed an additional experiment; where 2-year-old seedlings were inoculated with the same isolate of *H. fraxineus* and wounded by cryospray under controlled conditions to analyse the candidate gene expression patterns during the early stages of the interaction.

## Results

### Trees with different levels of susceptibility display differences in expression patterns in both non-symptomatic and symptomatic tissues

Branches of four trees with known levels of susceptibility (susceptible clones S21K926076 and S21K916009 and resistant clones S21K926100 and S21K916008 [22]) were inoculated with *H. fraxineus*. Ten months after the inoculation the discernible necrosis was at least three times as long in the previously established susceptible genotypes than in the resistant genotypes (Table 1, *P* < 0.01, Student’s t-test).

**Table 1:** The lesion length in the barks of the ash clones 10 months after inoculation with *H. fraxineus* in the common garden at Trolleholm

ID	clones	Lesion length (cm)
S21K926100	Resistant	7
S21K916008	Resistant	8.5
S21K926076	Susceptible	32
S21K926009	Susceptible	70

Total RNA was extracted from the bark samples collected at, and distal to, the developed lesion and mRNA enriched samples were sequenced with Illumina Hiseq 2000. After quality filtering, the reads were aligned to the Ash genome BATG-0.4v3 (http://www.ashgenome.org) using the of *TopHat-Cufflinks* pipeline [23, 24]. After aligning filtered read data from each sample to the ash genome, in total 87,413 transcripts were presented in the libraries. The average read mapping frequency was 80% and the average of aligned pairs was 7,086,883 per library (Supplementary file S1). To estimate the amount of *H. fraxineus* in the samples the reads were also mapped to the *H. fraxineus* Nf4 genome (Elfstrand et al. in prep). The read mapping frequency varied from 0.1 to 6.6% among the samples (Supplementary file S2). No significant differences in the estimated *H. fraxineus* biomass were found between genotype categories or treatments, and the estimated biomass did not correlate with the length of the necrosis between genotype categories (Supplementary file S2).

### Developing necrosis is associated with substantial transcriptional changes

**Fig. 1:**
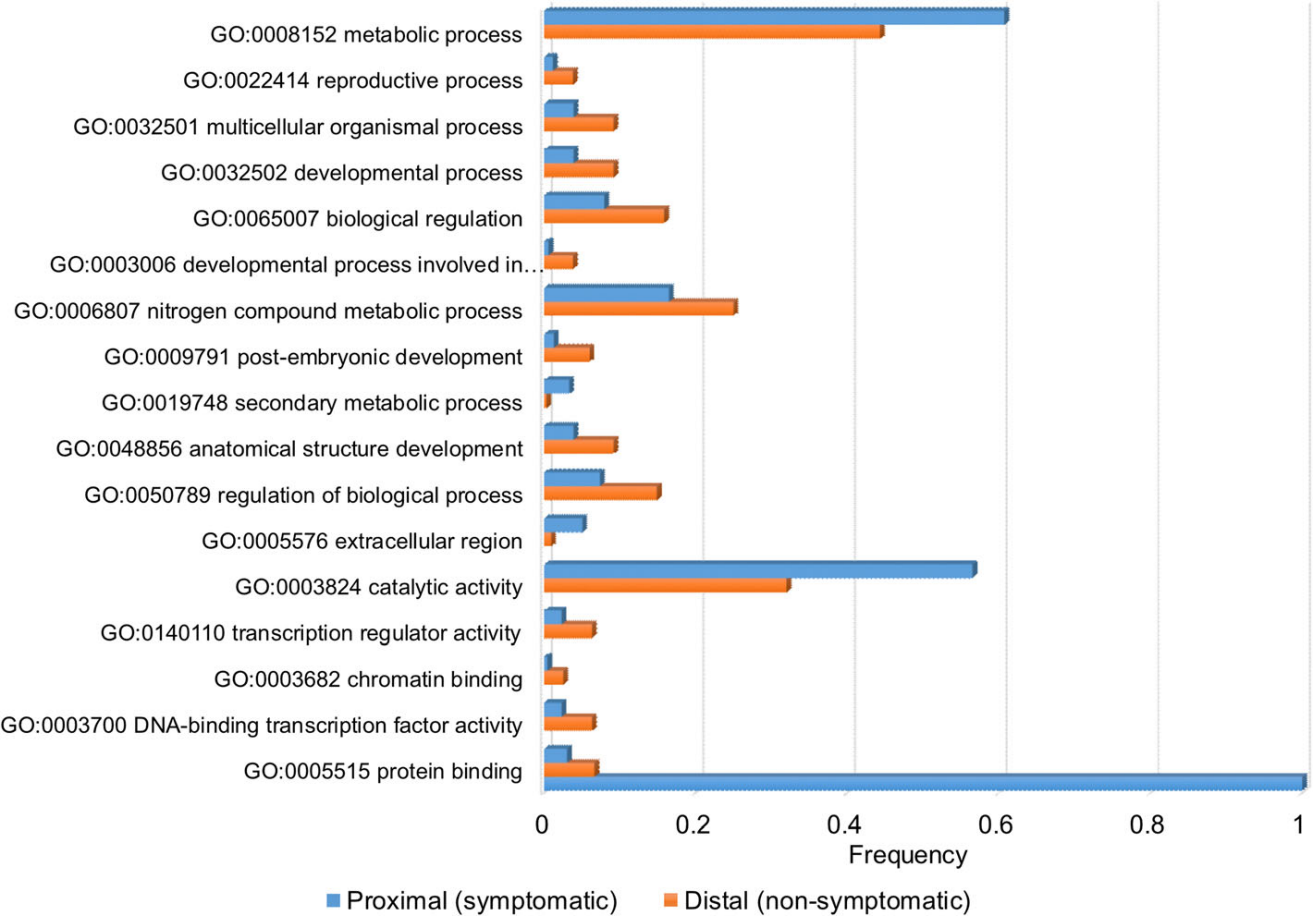
Significantly enriched GO terms in proximal symptomatic tissues (blue bars) and distal non symptomatic tissues (orange bars). The analysis is based on the DEGs presented in Table 2

The analysis of differential expression between proximal tissues inoculated with *H. fraxineus* and distal non-inoculated tissues showed that there was 1009 differentially expressed genes (DEGs) in the comparison between the proximal symptomatic and the distal non-symptomatic tissues. The majority of these DEGs (693) were more highly expressed in tissues adjacent to the necrosis (Table 2, Supplementary file 3). It is also noteworthy that we found 66 de novo expressed genes in symptomatic tissues compared to one single gene specifically expressed in distal tissues (Table 2). Among the genes de novo expressed in near symptomatic tissues were one *wall-associated receptor kinase 5* like gene (XLOC_046826_TCONS_00081726), while another *wall-associated receptor kinase* (XLOC_019616_TCONS_00033792) was found to be induced in the same contrast. A *Serine protease inhibitor, potato inhibitor I-type family protein* like gene XLOC_080002_TCONS_00138225 and five DEGs with similarity to *polygalacturonase* genes were also found to be de novo expressed in these samples (Supplemental file 3). The analysis of the GO terms associated with the DEGs showed that GO:0005576 extracellular region, GO:0003824 catalytic activity and GO:0008152 metabolic process were enriched in samples adjacent to the necrotic lesion compared to samples collected more distal (Fig. 1).

**Table 2:** Differentially expressed genes (DEGs) in proximal symptomatic and distal non-symptomatic bark of common ash after infection with *H. fraxineus* nf4 in the common garden experiment

Comparison	more highly expressed in symptomatic tissues		more highly expressed in non-symptomatic tissues		Total^c^
	DEG^a^	de novo^b^	DEG^a^	de novo^b^	
Symptomatic/non-symptomatic	693	66	316	1	1009

^a^the total number of DEGs which is more highly expressed in a given genotype category, this number includes the de novo expressed transcripts

^b^the number of the DEGs which is de novo expressed, unique, transcripts

^c^The total number of DEGs in the comparison

#### Transcriptional differences between resistant and susceptible genotype categories are less pronounced in symptomatic tissue

By comparing expression patterns in non-symptomatic tissues, we found 1082 DEGs which differed in their expression pattern between resistant and susceptible genotypes. About a third (329 DEGs) was more expressed in the resistant genotypes (Table 3). In the comparison of expression patterns in samples taken on the margin of the necrosis from genotypes categorized as resistant or susceptible only 138 DEGs were found, and 124 of these were de novo expressed in either genotype category (Table 3, Supplementary file 3). The uniquely expressed DEGs were on average shorter and less expressed (as judged by the count data in Supplementary file 3) than the DEGs in general. The vast majority of these transcripts were also de novo expressed when comparing non-symptomatic tissues of resistant and susceptible genotypes. Taken together, the results suggest that these transcripts represent genes differentially used between the genotype categories irrespective of treatment.

**Table 3:** Differentially expressed genes between trees in the resistant (R) and susceptible (S) categories in proximal symptomatic and distal non-symptomatic bark of common ash after infection with *H. fraxineus* nf4 in the common garden experiment

Comparison	More highly expressed in R		More highly expressed in S		Total^c^
	DEG^a^	de novo^b^	DEG^a^	*de novo*^b^	
R (Non-symptomatic)/ S (Non-symptomatic)	329	165	753	93	1082
R (symptomatic)/ S (symptomatic)	76	73	62	51	138

^a^the total number of DEGs which is more highly expressed in a given genotype category, this number includes the *de novo* expressed transcripts

^b^the number of the DEGs which is *de novo* expressed, unique, transcripts

^c^The total number of DEGs in the comparison

A gene that we consider relatively interesting in the comparison between resistant and susceptible genotypes is XLOC_074973_TCONS_00129820 a *probable leucine-rich repeat receptor-like serine threonine-protein kinase*, because this transcript appears to be turned off in symptomatic tissues in the susceptible genotypes (Supplementary file 3). It is noteworthy that we found 12 different *TIFY/JAZ* DEGs in our dataset and that seven of these show maximum expression in non-symptomatic tissues in the susceptible genotypes (Log 2-fold change was)0.9–3.7 compared to the resistant genotypes (Supplemental file 3).

#### Two-way clustering of DEGs reveal co-expressed genes in the mevalonate (MVA) and iridoid pathways

**Fig. 2:**
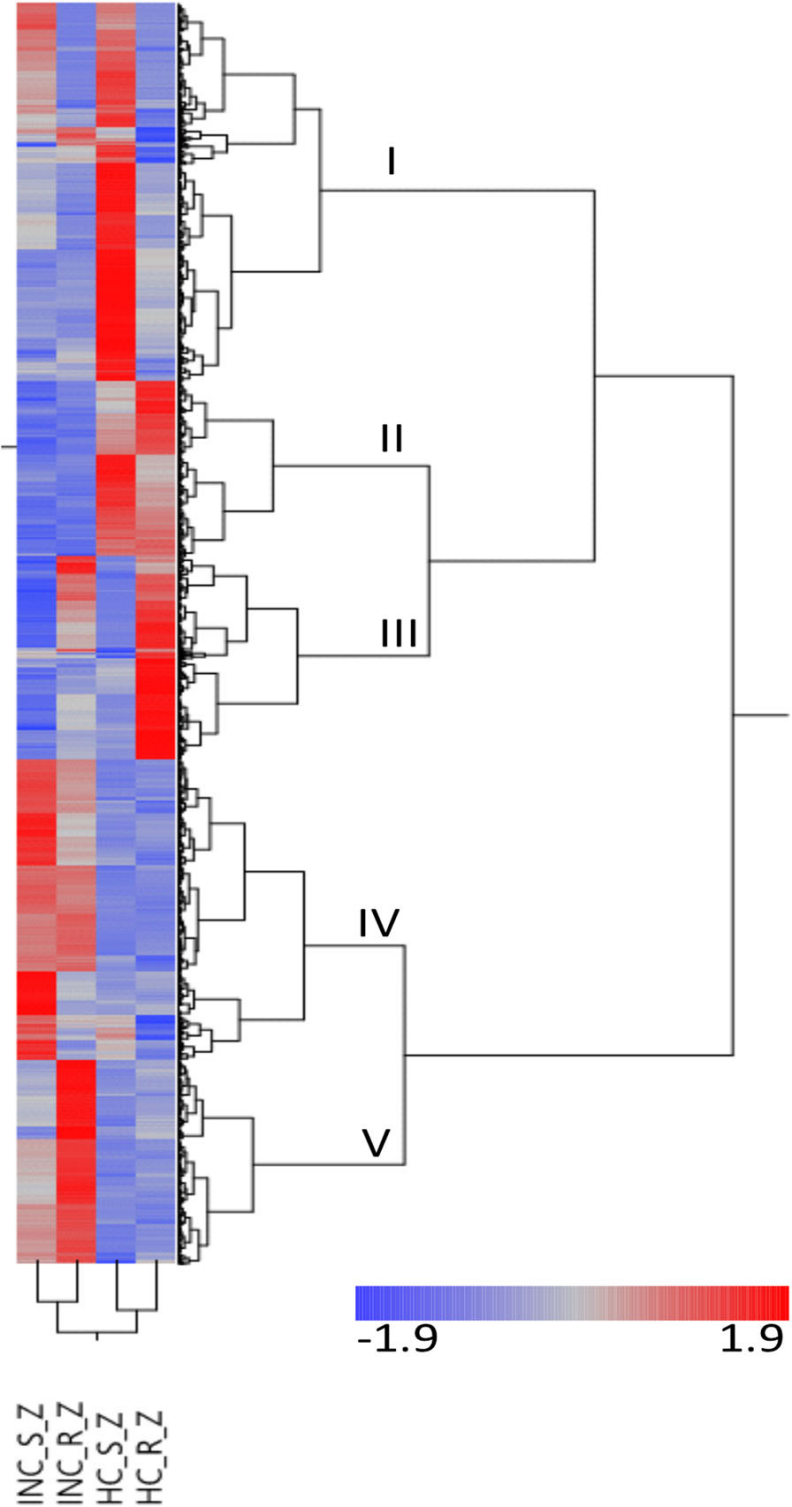
A two-way hierarchical clustering of the DEGs that were differentially expressed in at least one comparison. INC_S_Z and HC_S_Z, represents the expression levels in in proximal symptomatic tissues and distal non-symptomatic tissues respectively in samples from the susceptible genotype category. Similarly, INC_R_Z and HC_R_Z, represents the expression levels in in proximal symptomatic tissues and distal non-symptomatic tissues respectively in samples, but in the resistant genotype category. The heatmap represent Z-scores. The co-expression clusters are indicated with Roman numbers and letters. In-data is found in Supplementary file S3

A two-way hierarchical clustering of the genes that were differentially expressed in at least one comparison revealed five co-expressed clusters (Fig. 2). The first cluster (I) comprised genes that show their highest expression level in apparently healthy distal tissues in susceptible trees. Out of 607 genes in cluster I, 595 are differentially expressed between the two genotype categories in healthy tissues (Supplementary file 3). The GO term enrichment analysis shows that cluster I is enriched for the terms *kinase activity* (GO:0016301), *heterocyclic compound binding* (GO:1901363) and *organic cyclic compound binding* (GO:0097159). The genes in cluster II are significantly less expressed in samples taken proximal to lesion than in distal asymptomatic tissues (Fig. 2, Supplementary file 3). The cluster is enriched for genes associated with eg. the GO terms *transport* (GO:0006810), *cell-cell signaling* (GO:0007267), *shoot system development* (GO:0048367) and *developmental process involved in reproduction* (GO:0003006). Several genes associated with auxin transport and auxin-mediated transcriptional activation/repression (eg. XLOC_007030_TCONS_00012066, XLOC_082174_TCONS_00141728, XLOC_045923_TCONS_00080158 and XLOC_083592_TCONS_00144115) are found in this cluster (Supplementary file 3). The genes in cluster III are most highly expressed in apparently healthy distal tissues in resistant trees (Fig. 2). Just like in cluster I, the vast majority (94%) of the genes in cluster III are differentially regulated between the two genotype categories in distal healthy tissues, but not in other contrasts. Clusters IV and V include genes which are more highly expressed proximal to the lesion than in the distal healthy-looking tissues (Fig. 2). The enrichment analysis of the GO terms associated with the clusters showed that cluster IV was enriched for, among others, the GO terms *secondary metabolic process* (GO:0019748), *intracellular* (GO:0005622) and *hydrolase activity* (GO:0016787) while cluster V was enriched for the GO term *carbohydrate metabolic process* (GO:0005975). (Supplementary file 3). Quite many of the genes in cluster IV (118 of 483 genes) were also significantly more active in distal, seemingly healthy tissues in susceptible trees but not from resistant trees (Supplementary file 3).

Cluster IV, which was enriched with the GO term *secondary metabolic process* (GO:0019748), harbours 19 DEGs encoding enzymes in the phenylpropanoid, stilbenoid and flavonoid biosynthetic pathways (Supplementary file 3). The cluster also includes seven DEGs in the mevalonate (MVA) pathway producing precursors of terpenoids, sterols and isoprenoids; *HMGS* (*hydroxymethylglutaryl-synthase-like*, XLOC_023247_TCONS_00040192), *HMGR1* (*3-hydroxy-3-methylglutaryl-coenzyme a reductase 1*, XLOC_038797_TCONS_00067684), *IDI* (*isopentenyl-diphosphate delta-isomerase*, XLOC_013079_TCONS_00022546), MVK (Mevalonate kinase XLOC_036719_TCONS_00064121 and XLOC_060762_TCONS_00105621) and *FPPS/FPS* (*farnesyl diphosphate synthase*, XLOC_029273_TCONS_00050861 and XLOC_021137_TCONS_00036448). The *HMGR1* gene model is one of the most strongly differentially expressed genes in the comparison between symptomatic proximal tissues and distal non symptomatic tissues, as it is upregulated by 8.8 times.

It appears that the monoterpenoid and iridoid biosynthesis pathways may be co-regulated with the MVA pathway proximal to the lesion as genes encoding enzymes in the monoterpenoid (XLOC_012295_TCONS_00021196 and XLOC_070583_TCONS_00122368) and iridoid biosynthesis pathways (XLOC_015937_TCONS_00027469, XLOC_054974_TCONS_00095590, XLOC_057453_TCONS_00099966 and XLOC_086030_TCONS_00148326) (Supplementary file 3) also cluster in cluster IV. Interestingly, the bHLH transcription factor XLOC_049531_TCONS_00086405 incluster IV (Supplementary file 3) is a putative *Fraxinus* ortholog of the *bHLH iridoid synthesis (BIS)* genes. The BIS transcription factors specifically controls the genes in the iridoid biosynthesis pathway in *Catharanthus roseus* [25, 26].

### The transcriptional responses bordering necrosis are similar in early- and late phases of infection

To investigate if the responses seen at the border of a fully developed necrosis reflects the early resistance responses we conducted an inoculation experiment on two-year-old plants under controlled conditions in the greenhouse, comparing healthy, cryo-wounded, and *H. fraxineus*-inoculated samples after 2 weeks.

Following RNA sequencing of samples and mapping reads to the ash genome (Supplementary file S4), 70,083 predicted ash transcripts were represented in the RNAseq libraries. The locus information from the transcripts mapped in the *TopHat-Cufflinks* pipeline were joined to with the information obtained from the earlier common garden experiment to allow for comparisons of DEGs.

**Fig. 3:**
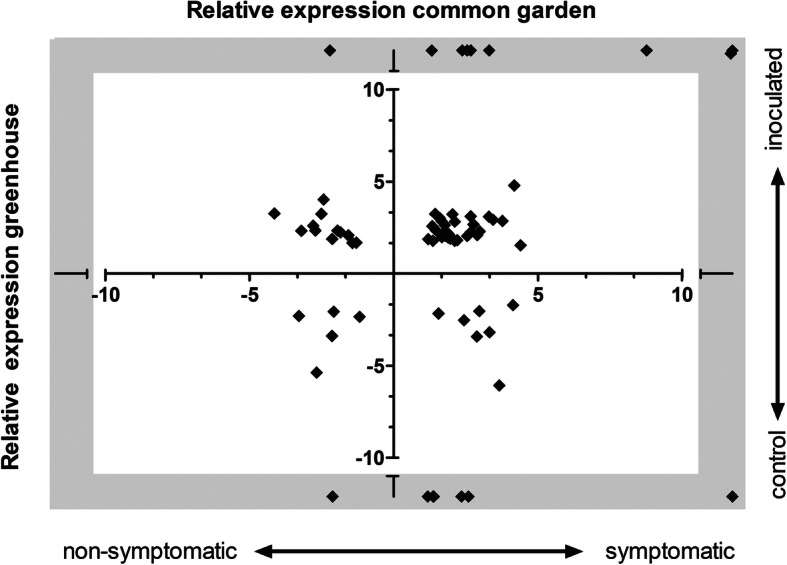
Scatter plot of concordant and discordantly expressed DEGs between the common garden and green house experiments. On the horizontal axis the relative expression level ((log2) fold change) of the genes in the common garden experiment are plotted, positive values indicate higher transcript accumulation levels in symptomatic tissues and negative values represents higher expression in non-symptomatic tissues. The grey area shading the interrupted axis indicate genes which were specifically (*de novo*) expressed in one of the samples in the experiment. The greenhouse experiment is plotted on the vertical axis, again positive values indicate higher transcript accumulation levels (positive (log2) fold change values) in symptomatic tissues and negative values represents higher expression in control samples

In the two comparisons involving *H. fraxineus* inoculation (i.e. control vs inoculation and wounding vs inoculation), 204 and 151 DEGs were found, respectively. Comparing control vs inoculation, 149 of the DEGs were induced and 55 repressed after inoculation. When comparing inoculation to wounding treatment, 87 DEGs were induced and 64 were repressed. In total, 318 transcripts showed differential expression at the border of the necrosis at 14 dpi. Seventy-two of these DEGs were identical to DEGs samples collected nine-months after inoculation in the common garden experiment (Fig. 3, Supplementary Table S5). Two-thirds (47 genes) showed similar expression pattern in the two experiments (Fig. 3, Supplementary Table 5) and the vast majority are up regulated adjacent to the necrosis. For instance, the transcript XLOC_080002_TCONS_00138225, encoding a serine proteinase inhibitor, is de novo expressed adjacent to the necrosis compared to seemingly healthy tissues in both field conditions and greenhouse conditions. The previously mentioned *HMGR1* gene (XLOC_038797_TCONS_00067684) is also induced adjacent to the necrosis in both experiments together with the downstream gene XLOC_027474_TCONS_00047699 (*squalene epoxidase 2-like, SQE2*). XLOC_060634_TCONS_00105428 which appears to be an ortholog to the *Arabidopsis* NAC transcription factor *ATAF1* is also up-regulated in both studies (Supplementary Table 5). Among the few genes that were suppressed proximal to the necrosis in both experiments we found XLOC_065346_TCONS00113529 with significant similarity to *Arabidopsis BT1* and *BT2* genes. Interestingly, several of the *TIFY* DEGs, encoding JAZ proteins, and transcripts encoding well-characterized regulators in the ABA signaling pathway HAT22 (XLOC_017277_TCONS_00029721), AHG3 (XLOC_035596_TCONS_00062118) and HAI2 (XLOC_020080_TCONS_00034570) were also present among the genes that were differentially expressed in both datasets (Supplementary Table S5). The *TIFY* DEGs, encoding JAZ1-like proteins, were upregulated proximally to the necrosis during this early phase of the interactions, but after 10 months’ interaction, the DEGs were downregulated proximally to the necrosis. Furthermore, these DEGs had significantly higher expression in distal non-symptomatic tissues of susceptible genotypes compared to resistant genotypes (Supplementary file 3).

## Discussion

Ten months after the inoculation with *H. fraxineus* on two susceptible and two resistant ash genotypes the extension of the necrosis was at least three times as long in the susceptible genotypes than in the resistant genotypes. An analysis of the fraction of *H. fraxineus* reads in the RNA seq libraries showed that there was no correlation between lesion extension, or proximity to the lesion, and *H. fraxineus* biomass in the tissues. Thus, we opted to call the samples taken on the margin of the necrosis samples from “proximal/symptomatic” and the samples taken from apparently healthy tissues distally to the necrosis “distal/non-symptomatic” much like in the study by Kovalchuk and collaborators [27]. We found 1009 genes that were differentially expressed between the proximal, symptomatic and the distal, non-symptomatic samples showing that the developing necrosis in the bark and the phloem is associated with substantial transcriptional changes leading up to e.g. the metabolic changes that are associated with necroses in ash [8, 28].

The significantly different extension of the necrosis between susceptible and resistant genotype categories were accompanied by the differential expression of 138 transcripts between the genotype categories in proximal symptomatic tissues and 1082 in the distal, non-symptomatic tissues. This can be interpreted such that the difference in necrosis extension between trees with different levels of susceptibility is associated with differences in expression patterns in both non-symptomatic and symptomatic tissues, i.e. trees with different levels of resistance show different basal levels of gene expression and possibly different transcriptional responses proximal to the advancing necrosis. However, among the 138 DEGs in symptomatic tissues from resistant and susceptible genotypes, the vast majority (> 100 DEGs) were also specifically (de novo) expressed in either category of trees independent of their proximity to the inoculation. These results indicate that these DEGs may represent structural copy number variation (CNV) in the genomes or genes differentially used between the genotype categories irrespective of treatment [29, 30].. Among the DEGs which were specifically expressed in either genotype category in the proximal samples but expressed in both genotype categories in distal non-symptomatic tissues we identified a transcript from a *probable leucine-rich repeat receptor-like serine threonine-protein kinase* gene which clearly had higher expression in the resistant category. The transcript in question was however expressed more in the non-symptomatic tissues and in particular, in non-symptomatic tissues in resistant trees, indicating that this particular transcript represents a gene differentially used between the genotype categories. However, to understand if these uniquely expressed transcripts, and in particular those that show identical responses in both proximal and distal samples, are representing CNVs or actual DEGs the current analysis would need to be complemented with genetic mapping or whole genome resequencing.

The two-way clustering identified a cluster of DEGs that were upregulated on average 2.6-fold in the symptomatic tissues proximal to the necrotic lesion, indicating that this cluster may be associated with controlling the spread of *H. fraxineus* and the developing necrosis. This cluster highlighted the co-regulation of genes in the MVA (*HMGS, HMGR1, MVK, IDI* and *FPPS/FPS*) and iridoid (eg. the *7-deoxyloganetic acid glucosyltransferase* gene) pathways. The cytosolic MVA pathway produces isopentenyl diphosphate (IPP) and dimethylallyl diphosphate (DMAPP) precursors for isoprenoids such as terpene-, sterol- and steroid biosynthesis in plants, HMGR1 is considered the rate-limiting enzyme that control the flux through the pathway [31–34]. It is established that infection with *H. fraxineus* lead to accumulation of phenolic and isoprenoid compounds in ash [8, 28]. The susceptibility of ash trees to *H. fraxineus* have been associated to iridoid glycoside levels in leaf tissues; highly susceptible genotypes displayed higher levels of several different iridoid glycosides than resistant genotypes [7, 9]. It has been postulated that in the Oleaceae family the monoterpene derivative deoxyloganic acid is likely to be an intermediate in the biosynthesis of secoiridoid and iridoid glycosides [35]. Although we did not quantify the levels of secondary metabolites in this study, it likely that the transcriptional activation of genes encoding enzymes in the phenylpropanoid, terpenoid and iridoid pathways is reflected in the accumulation of phenolics and terpenes, iridoids and other isoprenoids proximal to the developing necrosis. The activation of *HMGR1* and *SQE2* in proximal symptomatic tissues compared to the control samples in both experiments in this study underlines that increased fluxes through the MVA pathway are likely to be associated with controlling the spread of *H. fraxineus* and the resulting necrosis in lesions in ash during several phases of the interaction.

By comparing the transcriptional responses in the common garden experiment to an experiment performed under controlled conditions in the greenhouse we attempted to shed light on the defence responses in ash to *H. fraxineus* infection, assuming that genes consistently expressed between experiments are associated with defence to the pathogen*.* We found a relatively small set of genes that were differentially expressed in both experiments, including the previously discussed *HMGR1*. Given the vastly different experimental conditions this is not unexpected, rather it supports the possibility that the identified co-regulated genes are associated to the interaction. One of the de novo expressed transcripts is a serine proteinase inhibitor in the PR-6 family. It has been reported previously that serine proteinase inhibitor activity and transcript levels increase in *Fraxinus* spp. during feeding of tissues by Emerald ash borer and when treated with Methyl Jasmonate (MeJA) [13, 36]. Proteinase inhibitors bind to proteinases and control proteinase activity, a function needed to modulate multiple processes *in planta* including defence to attackers. In plant-fungal interactions plant proteinase inhibitors may for instance reduce the ability of the fungus to use its proteinases necessary for pathogenicity [37]. The de novo expressed ash proteinase inhibitor is similar to the *Arabidopsis UNUSUAL SERINE PROTEASE INHIBITOR* (*UPI*) gene. *UPI,* which is induced by Jasmonate (JA), Salicylic acid (SA) and abscisic acid (ABA), and is a component of the *Arabidopsis* response to necrotrophic fungal infection and insect herbivory [38]. Laluk and Mengiste [38] propose that UPI may be involved in the containment of the necrosis during necrotrophic infection blocking cell death induced by fungal toxins. It is easy to envision the need for ash to attempt to control cell death and necrosis progression in the interaction with *H. fraxineus*.

Several of the expressed candidate genes show significant similarity to genes encoding players in the ABA- or JA-signalling pathways. We have previously reported that ash seedlings from both the resistant and susceptible genotype categories develops necroses and accumulate intermediates in the ABA biosynthesis pathway associated upon treatment with the phytotoxin viridiol produced by *H. fraxineus* [8]. ATAF1 and several of its paralogs in *Arabidopsis* are important transcriptional integrators between abiotic [39–41] and biotic [41–43] stress. ATAF1 regulates several genes in the ABA metabolism including *NCED3* encoding the key ABA biosynthetic enzyme 9-cis-epoxycarotenoid dioxygenase [44, 45], so it is possible that activation of the ash ATAF1 ortholog proximal to the necrosis activates the ABA biosynthesis pathway in the tissue. The observation that orthologs of the Arabidopsis transcription factor *HAT22* and of the PP2C gene *AHG3* show moderate upregulation in both experiments, and the flux through the MVA pathway supports the interpretation that ABA accumulates also in response to necroticisation by the living pathogen. Both HAT22 and AHG3 are central players in the ABA signalling cascade; HAT22 regulates the expression of, among others, *AHG3* in *Arabidopsis* in response to ABA treatment and abiotic stress [46, 47]. In contrast, JA signalling appear to be differentially activated proximal to the necrosis in the two datasets; in the common garden experiment JA biosynthesis genes such as *OPR3* and *lipoxygenase* are upregulated proximal to the lesion, while JAZ/TIFY proteins are down regulated. However, the role of JA-signalling in the interaction may be complex, four transcripts with significant similarity to *JAZ1 (TIFY)* show contrasting regulation between the two experiments. JAZ proteins are among the most important components of the JA pathway, acting both as co-receptors of the hormone, together with the COI1 receptor, and as repressors of downstream TFs modulating JA-induced defence responses in plants [48]. The induced expression in the early interaction of JAZ proteins suggests that JA signalling is downregulated proximal to the necrosis during this phase of the interaction. *JAZ/TIFY* genes are among the DEGs that are regulated between the genotype categories in the common garden experiment. Their expression pattern suggest that the JA pathway is more active in the resistant trees than in susceptible trees and that JA-mediated responses may be important in controlling the spread of *H. fraxineus* in developing lesions on ash.

**Fig. 4:**
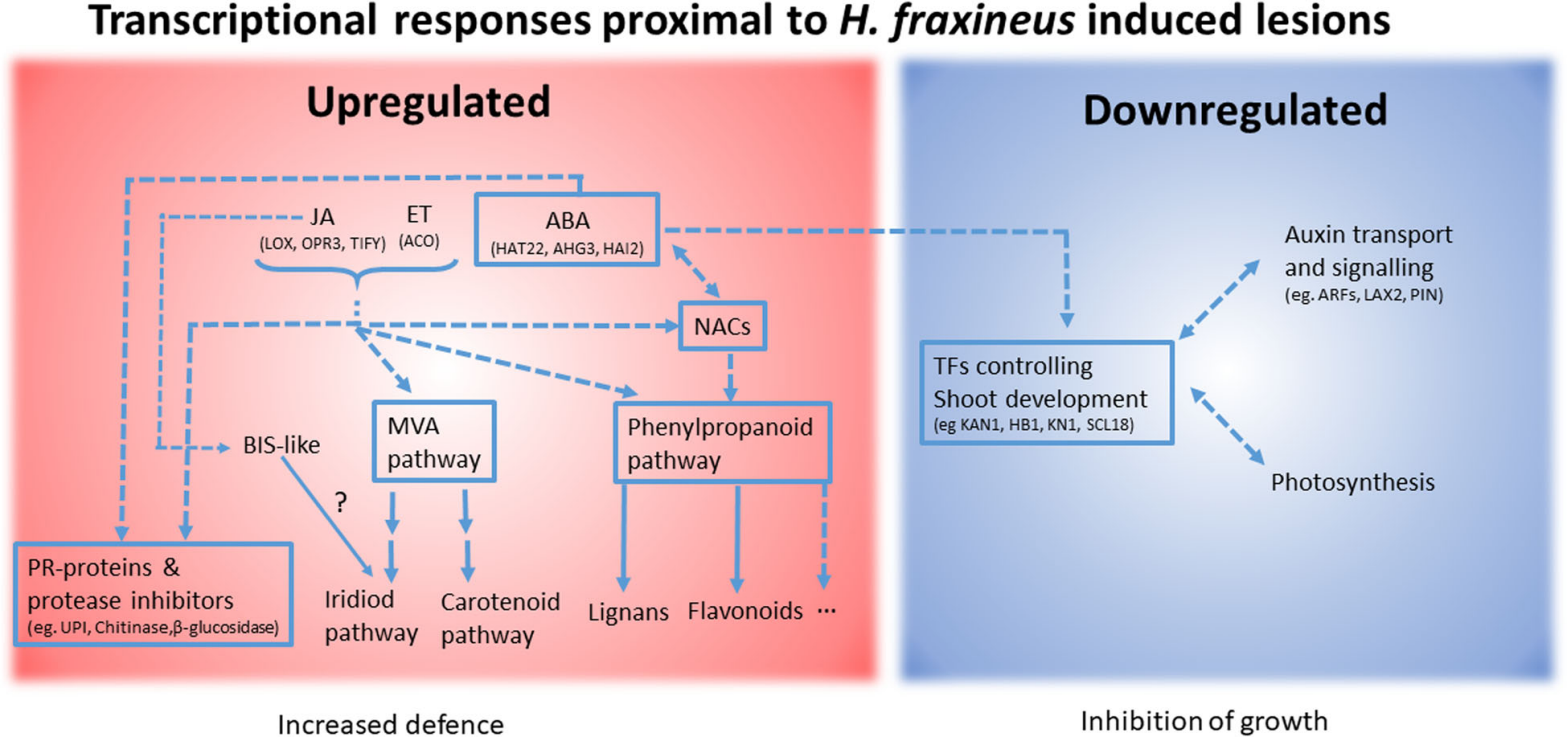
Tentative model of the transcriptional responses in *F. excelsior* to *H. fraxineus* inoculation in the phloem. The red field indicate signaling pathways and metabolic pathways that are significantly upregulated proximal to the lesion. The blue field indicate signaling, developmental and metabolic pathways that are downregulated proximal to the lesion. Pathways or gene categories surrounded by a frame are consistently regulated in both early and late phases of lesion development. Arrows indicate detected co-regulation of DEGs and pathways and dashed lines suggest relationships between pathways and DEGs

In conclusion we could show that the differences in necrosis extension seen between susceptible and resistant genotype categories were not associated with differences in *H. fraxineus* biomass and associated to relatively limited differences in DEG patterns between genotype categories at the necrosis front. Despite this, we could identify a set of candidate genes, hormone signalling pathways and secondary metabolite biosynthesis pathways (Fig. 4), which are likely to be involved in the containment of the necrosis after inoculation with *H. fraxineus*.

## Methods

### Inoculation experiments

#### Common garden inoculation experiment

Branches of two susceptible genotypes (clones S21K926076 and S21K916009) and two resistant genotypes (clones S21K926100 and S21K916008) of European ash growing in a common garden at Trolleholm in southern Sweden (55.937076, 13.282873) [22] were artificially inoculated with *H. fraxineus* (isolate nf4, previously collected from an infected ash tree in Sweden). The trees and the site are part of a Swedish regional ash breeding program at SkogForsk, and the experiment was carried out with Skogforsk’s permission. The experimental design and sampling procedure was as follows; healthy-looking branches were selected in September 2011, a bark flap was cut with a scalpel, and inoculated with agar plugs from two-week-old *H. fraxineus* cultures growing on 2% malt extract agar and sealed with parafilm and another nylon ribbon. After 10 months, in June 2012, the trees were sampled. At sampling, the extension of the discernible lesion in the inner bark was measured from the inoculation site and the extent of visible necrosis in the wood was estimated following longitudinal section of the branches. Three samples (ca 2 × 0.5 cm) were collected the edge of the discernible lesion on each of the four clones. Three samples from non-inoculated and non-symptomatic branches of the same clones were taken as controls. The excised bark samples were immediately immersed in RNA*later*™ Stabilization Solution and stored at − 20 °C until extractions could be performed.

#### Greenhouse inoculation experiments

Two-year-old bare-root seedlings of *F. excelsior*, 30–50 cm in height, were obtained from a commercial nursery near Helsingborg, Sweden in March 2011. The ash seedlings were planted in plastic pots (20 cm diameter) filled with light sieved peat, black peat and sand in 60:25:15 proportion, respectively (Hasselfors garden, Örebro, Sweden). The plants were kept in a greenhouse with a 16 h photoperiod, 20/15 °C (day/night) temperature, and watered as needed for 7 weeks before being subjected to one of the following treatments: 1) healthy control; 2) wounding and 3) inoculation with *H. fraxineus*. Wounding was performed by applying a 5 mm sponge soaked in a liquid cryospray (Cryospray 59, Cuxson Gerrad, UK) to the bark surface for 10 s. Inoculum was prepared by adding sterile wood plugs to 2-week-old cultures of *H. fraxineus* (nf4). After 4 weeks, plugs fully colonized by *H. fraxineus* were used to inoculate ash seedling by cutting a 1 × 1 cm section of bark and applying the inoculum plug directly to the vascular cambium using sterilized forceps, and then sealing the wound with Parafilm. Macroscopic observations of lesion development were made at 7, 14, 28 and 42 days post-inoculation. At each sampling date, seedlings were destructively sampled and samples were collected from all treatments. Phloem tissue was collected from the freezing injury or point of inoculation by removing a 1 × 2 cm section with a sterile scalpel. Tissue was then frozen in liquid N_2_ and stored at -80C until RNA extraction. For the current study, we examined only those samples collected 14-days after wounding and inoculation treatments.

### RNA extraction, cDNA synthesis and sample preparation

After total RNA extraction [49], samples were treated with DNaseI (SIGMA) to remove the genomic DNA before being stored at − 70 °C. The RNA concentration was measured on a BioAnalyzer 2100 (Agilent). Dynabeads® mRNA Purification Kit (Invitrogen) was used according manufacturer specifications, to extract poly(A) + RNA from samples. Messenger RNA amplification was conducted according to the manufacturer’s instruction using MessageAmpIII kit (Ambion). Then, from the amplified RNA (aRNA), cDNA was synthesized using iScript cDNA Synthesis Kit (Bio-Rad) according to the manufacturer’s specification with one exception: that the RT-reaction was continued for approximately 50 min. The second strand cDNA was then synthesized according to the protocol described by Sambrook and Russel [50]. This was followed by pooling enough quality of double stranded cDNA according to treatments. Between 2 and 5 μg from each cDNA samples were then sent to SciLifeLab (Stockholm) for library preparation using Illumina TruSeq and sequencing (2 × 100 bp) on an Illumina HiSeq 2000 instrument.

### Bioinformatics and statistics

The reads obtained from the Illumina sequencing was filtered with Nesoni (http://www.vicbioinformatics.com/software.nesoni.shtml) to remove low quality bases (quality below 20), adaptor sequences, and reads shorter than 55 bp after trimming. The filtered read data was aligned to the Ash genome BATG-0.4v3 (http://www.ashgenome.org) using the of *TopHat-Cufflinks* pipeline [23, 24], using recommended settings. Thereafter, *Cuffdiff* was used to identify genes that were differentially regulated in each treatment, using reads and merged assemblies, in accordance with the recommended settings. To visualize the results from the *TopHat-Cufflinks* pipeline we used both *CummeRbund* [23, 24] and JMP Pro 13 (SAS institute). To estimate the amount of *H. fraxineus* in the samples the filtered read data were aligned to the *H. fraxineus* Nf 4 genome (Elfstrand et al. submitted MS) using *Tophat* [23, 51] and the fraction of *H. fraxineus* reads in each library was estimated ((mapped reads/total reads in library) × 100). The FASTA sequence of the DEGs are available in Supplementary file S6.

Annotations of DEGs were accessed from the Ash Tree genome homepage (http://www.ashgenome.org) when available and complemented with manual annotations using the Blast2GO software suite [52, 53] when annotations were missing. We also used Blast2GO to compare GO term enrichment in different comparisons using the Fischer exact test, (with a cut-off of FDR < 0.05) and KEGG pathway mapping.

In order to make a two-way clustering of the expression patterns of genes that were differentially expressed in at least one of the comparisons made, the FPKM values in the treatments INC_S (proximal symptomatic tissues from the susceptible trees) and HC_S, (distal non-symptomatic tissues in samples from the susceptible trees) and INC_R and HC_R (the corresponding treated and non-treated samples the resistant trees) were converted to Z-scores,. The Z-scores were imported into JMP Pro 13 and a two-way hierarchical clustering, was made using *Ward’s method* (data provided in Supplementary file S3). The expression patterns were then visualized with a heatmap.

The samples from the common garden and green house experiments were analysed separately and the mapping data from the two experiments was joined using an in house python script to allow for comparisons of DEGs in the two experiments. The DEGs which were differentially expressed in both experiments were queried against the *Arabidopsis thaliana* v 11 proteome using the BLASTX algorithm. The FASTA sequence of these DEGs are also available in Supplementary file S6.

## Supplementary information

**Additional file 1. **Mapping results against the *F. excelsior* genome from the RNAseq libraries generated from the common garden experiment

**Additional file 2. **Estimation of *H. fraxineus* biomass in the common garden experiment using the fraction of reads mapping against the *H. fraxineus* genome in the RNAseq libraries. Average fraction of reads mapped to the *H. fraxineus* genome and the standard deviation are listed in the table.

**Additional file 3.** Annotation, cluster affiliation and relative expression levels of all DEGs in the common garden experiment

**Additional file 4.** Read mapping results and DEGs in the greenhouse experiment

**Additional file 5.** Concordant and discordantly expressed DEGs between the common garden and green house experiments

**Additional file 6.** The FASTA sequences of all DEGs detected in both studies

## Data Availability

The raw reads from the RNA sequencing experiments are deposited with the NCBI in PRJNA663173 and PRJNA663171.
