# Supplementary figures and images for: Transcriptional responses in developing lesions of European common ash (Fraxinus excelsior) reveal genes responding to infection by Hymenoscyphus fraxineus
Source: BMC Plant Biol. 2020 Oct 6;20:455. doi: 10.1186/s12870-020-02656-1 (PMC7541206; doi:10.1186/s12870-020-02656-1)

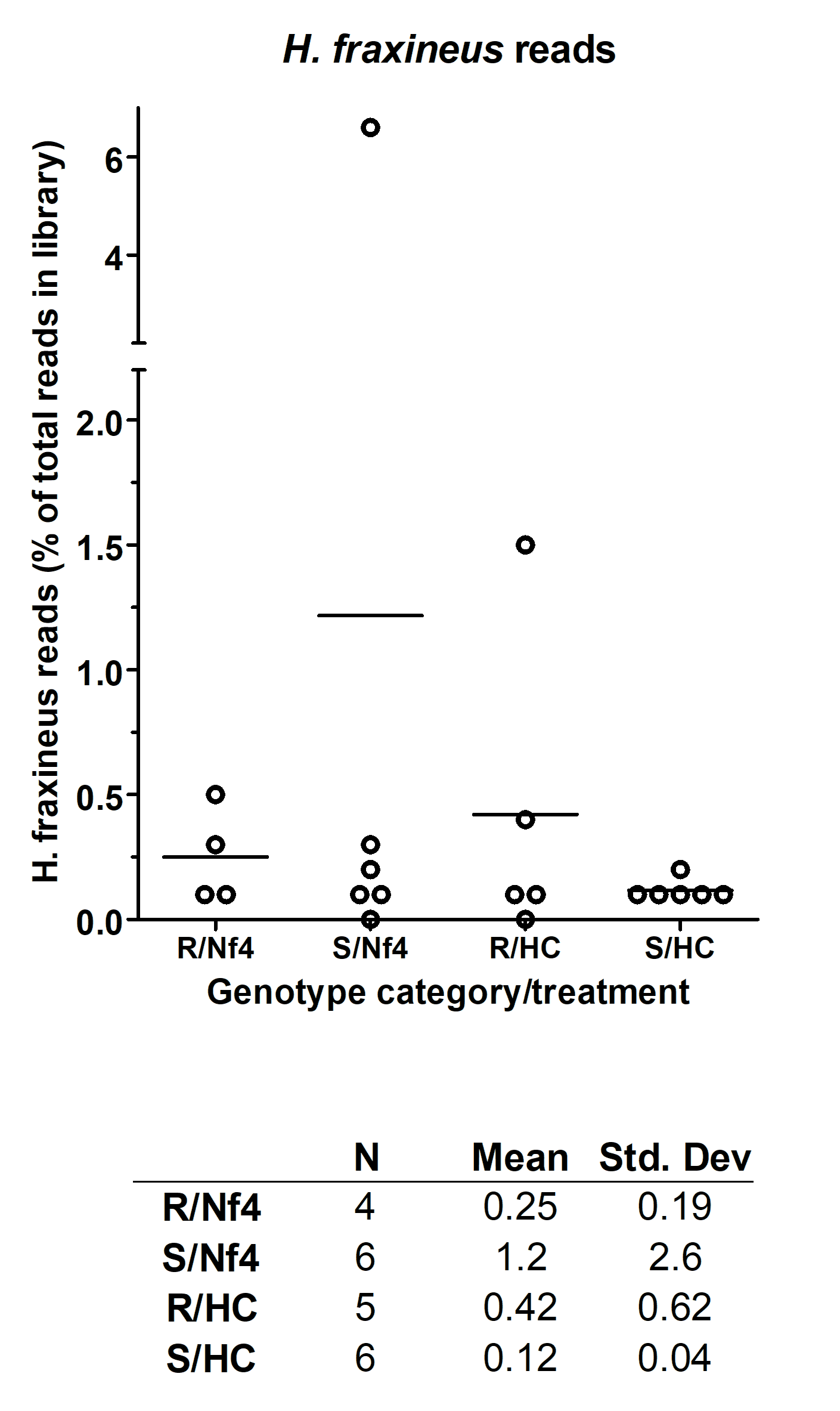

Supplement: Supplementary file 2 — Additional file 2. Estimation of H. fraxineus biomass in the common garden experiment using the fraction of reads mapping against the H. fraxineus genome in the RNAseq libraries. Average fraction of reads mapped to the H. fraxineus genome and the standard deviation are listed in the table. [file 12870_2020_2656_MOESM2_ESM.tif]
